# SeruNet-MS: A Two-Stage Interpretable Framework for Multiple Sclerosis Risk Prediction with SHAP-Based Explainability

**DOI:** 10.3390/neurolint17090151

**Published:** 2025-09-22

**Authors:** Serra Aksoy, Pinar Demircioglu, Ismail Bogrekci

**Affiliations:** 1 Institute of Computer Science, Ludwig Maximilian University of Munich (LMU), Oettingenstrasse 67, 80538 Munich, Germany; 2 Department of Mechanical Engineering, Aydin Adnan Menderes University (ADU), Aytepe, Aydin 09010, Türkiye; pinar.demircioglu@adu.edu.tr (P.D.); ibogrekci@adu.edu.tr (I.B.)

**Keywords:** SeruNet-MS, multiple sclerosis (MS), clinically isolated syndrome (CIS), two-stage machine learning, explainable AI, SHAP analysis, clinical decision support, demographic bias, predictive modeling, disease progression, neurological biomarkers

## Abstract

**Background/Objectives:** Multiple sclerosis (MS) is a chronic demyelinating disease where early identification of patients at risk of conversion from clinically isolated syndrome (CIS) to clinically definite MS remains a critical unmet clinical need. Existing machine learning approaches often lack interpretability, limiting clinical trust and adoption. The objective of this research was to develop a novel two-stage machine learning framework with comprehensive explainability to predict CIS-to-MS conversion while addressing demographic bias and interpretability limitations. **Methods:** A cohort of 177 CIS patients from the National Institute of Neurology and Neurosurgery in Mexico City was analyzed using SeruNet-MS, a two-stage framework that separates demographic baseline risk from clinical risk modification. Stage 1 applied logistic regression to demographic features, while Stage 2 incorporated 25 clinical and symptom features, including MRI lesions, cerebrospinal fluid biomarkers, electrophysiological tests, and symptom characteristics. Patient-level interpretability was achieved through SHAP (SHapley Additive exPlanations) analysis, providing transparent attribution of each factor’s contribution to risk assessment. **Results:** The two-stage model achieved a ROC-AUC of 0.909, accuracy of 0.806, precision of 0.842, and recall of 0.800, outperforming baseline machine learning methods. Cross-validation confirmed stable performance (0.838 ± 0.095 AUC) with appropriate generalization. SHAP analysis identified periventricular lesions, oligoclonal bands, and symptom complexity as the strongest predictors, with clinical examples illustrating transparent patient-specific risk communication. **Conclusions:** The two-stage approach effectively mitigates demographic bias by separating non-modifiable factors from actionable clinical findings. SHAP explanations provide clinicians with clear, individualized insights into prediction drivers, enhancing trust and supporting decision making. This framework demonstrates that high predictive performance can be achieved without sacrificing interpretability, representing a significant step forward for explainable AI in MS risk stratification and real-world clinical adoption.

## 1. Introduction

In recent years, numerous studies have sought to predict conversion from clinically isolated syndrome (CIS) to clinically definite multiple sclerosis (CDMS) by harnessing machine learning techniques on multimodal clinical and imaging data [1]. For example, Zhang et al. investigated 84 CIS patients drawn from a single-center database in Shanghai, extracting over 120 lesion shape and intensity features from baseline 3D FLAIR and T1-weighted MRI scans. A random forest classifier was trained on these features, achieving 84.5% accuracy, significantly outperforming conventional dissemination-in-space criteria, and highlighting that complex lesion morphology alone can serve as a robust predictor of CDMS conversion [2]. In a complementary approach, Wottschel et al. combined lesion load metrics (volume and count in periventricular, juxtacortical, and infratentorial regions) with demographic and basic clinical variables in a cohort of 112 CIS patients from two European centers. By employing a Support Vector Machine (SVM) with a radial basis function kernel, they demonstrated improved discrimination (AUC = 0.82) over standard radiological assessments, although interpretability was limited to global feature importance weights [3].

Subsequently, Ion-Mărgineanu et al. enriched MRI data with metabolic information derived from MR spectroscopy, specifically N-acetylaspartate and myo-inositol ratios, in a cohort of 96 CIS patients recruited at a tertiary care center in Bucharest. A logistic regression model incorporating both volumetric lesion measures and spectroscopy features yielded an AUC of 0.88, suggesting that microstructural biomarkers can augment predictive performance but at the cost of requiring specialized acquisition protocols [4]. Around the same time, Bendfeldt et al. employed a linear SVM on 68 CIS patients, using handcrafted descriptors of lesion shape, surface complexity, and spatial distribution as inputs; although overall accuracy reached only 78%, this work underscored the value of spatial lesion topology as an explanatory correlate of progression risk [5].

More recently, deep learning methods have been explored. Yoo et al. assembled a multi-center cohort of 212 CIS patients (across three North American hospitals), combining convolutional neural network features learned directly from FLAIR images with 25 clinical variables, including age, sex, oligoclonal band status, and initial symptom type. Their hybrid model achieved an AUC of 0.91, yet interpretability was limited to post hoc saliency maps, which offer only coarse localization of predictive regions without quantifying individual feature contributions [6]. In parallel, Daniel et al. evaluated five supervised classifiers, including random forest, XGBoost, and logistic regression, on a dual-site dataset comprising 411 CIS patients (138 Lithuanian and 273 Mexican). A random forest model attained a perfect F1-score on the Mexican subset (*n* = 273), with oligoclonal band positivity, spinal cord lesion presence, and abnormal evoked potentials surfacing as the top predictors; however, model transparency was limited to permutation importance rankings [7].

Haouam and Benmalek applied a finely tuned random forest to a cohort of 265 Mexican mestizo CIS patients, the same demographic focus as in the present work, yielding an AUC of 0.93 and 87% accuracy. Their pipeline relied on Recursive Feature Elimination to pare down 40 initial variables to 12, primarily imaging and immunological markers; however, threshold calibration and formal explainability beyond global feature rankings were not addressed [8].

Despite these innovations, key gaps remain in prevailing MS prediction methodologies. Algorithmic performance has been emphasized at the expense of clinical interpretability in most prior work, with global feature importance rankings or post hoc saliency maps alone reported that provide incomplete insight into patient-level predictions. Moreover, demographic baseline risk has not been delineated from modifiable clinical factors in traditional single-stage models, with potential introduction of demographic bias in clinical decision making and impairment of effective patient risk communication. Clinical implementation of AI-based MS prediction instruments has been impeded by the absence of transparent, case-level explanations, as insight into decision making processes is necessitated by neurologists to establish trust and incorporate recommendations into clinical workflows.

To overcome these limitations, SeruNet-MS is developed as a new two-stage MS risk prediction framework that formally decouples demographic baseline estimation from clinical risk refinement, paired with extensive SHAP-based explainability for per-patient predictions. Population-level demographic baseline risk is established first based on age, education, gender, and medical history, and then the baseline is refined based on clinical results, such as MRI lesion patterns, cerebrospinal fluid biomarkers, evoked potentials, and symptom characteristics. SHAP analysis is used to ensure transparent attribution of each factor’s contribution to per-patient predictions, allowing clinicians to see exactly which clinical variables are driving risk estimates. The system is validated on a cohort of 177 clinically isolated syndrome patients, with a ROC-AUC of 0.909 while retaining complete interpretability via real-time SHAP explanations. Through combination of demographic–clinical separation, state-of-the-art explainable AI, and practical clinical deployment, an interpretable framework for MS risk stratification is formalized that overcomes major interpretability limitations of previous studies while showing readiness for clinical deployment. Beyond its core role in risk prediction and clinical decision support, the two-stage framework also produces transparent outputs that can additionally serve educational purposes by showing how demographic and clinical factors shape individualized predictions. This educational use is a complementary benefit, while the primary motivation of the work remains clinical applicability.

## 2. Methodology

### 2.1. Data Acquisition and Preprocessing

This study used an open access dataset of a prospective cohort study carried out at the National Institute of Neurology and Neurosurgery (NINN) in Mexico City, Mexico, from 2006 to 2010. This dataset made available by Pineda and Flores Rivera (2023) with https://doi.org/10.17632/8wk5hjx7x2.1, accessed on 14 May 2025, and licensed under CC BY 4.0 through Mendeley Data. It includes extensive clinical and demographic data of Mexican mestizo patients with a new diagnosis of clinically isolated syndrome (CIS). The original cohort is a well-characterized sample of patients with their first clinical event with suspicion of central nervous system demyelination and is thus highly valuable in the study of conversion predictors to clinically definite multiple sclerosis (CDMS).

The original dataset included 273 patients and 20 clinical variables representing patient identifiers, demographic data, medical history, neurological presentation patterns, cerebrospinal fluid analysis, electrophysiological tests, and magnetic resonance imaging results. The variables were ID (patient ID), Age (age of the patient in years), Schooling (duration of schooling in years), Gender (1 = male, 2 = female), Breastfeeding (1 = yes, 2 = no, 3 = unknown), Varicella or chickenpox history (1 = positive, 2 = negative, 3 = unknown), Initial_Symptom (15 categories: 1 = visual, 2 = sensory, 3 = motor, 4 = other, 5 = visual and sensory, 6 = visual and motor, 7 = visual and others, 8 = sensory and motor, 9 = sensory and other, 10 = motor and other, 11 = visual, sensory, and motor, 12 = visual, sensory, and other, 13 = visual, motor, and other, 14 = sensory, motor, and other, 15 = visual, sensory, motor, and other), Mono_or_Polysymptomatic (1 = monosymptomatic, 2 = polysymptomatic, 3 = unknown), Oligoclonal_Bands (0 = negative, 1 = positive, 2 = unknown), LLSSEP or lower limb somatosensory evoked potentials (0 = negative, 1 = positive), ULSSEP or upper limb somatosensory evoked potentials (0 = negative, 1 = positive), VEP or visual evoked potentials (0 = negative, 1 = positive), BAEP or brainstem auditory evoked potentials (0 = negative, 1 = positive), Periventricular_MRI (0 = negative, 1 = positive), Cortical_MRI (0 = negative, 1 = positive), Infratentorial_MRI (0 = negative, 1 = positive), Spinal_Cord_MRI (0 = negative, 1 = positive), Initial_EDSS, Final_EDSS, and Group (1 = CDMS, 2 = non-CDMS).

A preprocessing pipeline was systematically enforced to guarantee data quality and analytical validity. Administrative variables and variables with 50% of the values or more missing were first excluded from the analysis. In particular, the unnamed index column (Unnamed: 0), Initial_EDSS scores, and Final_EDSS scores were dropped, as these variables were incomplete or not germane to the prediction task. Missing data handling then adhered to a structured process in which variables with explicit “unknown” categories were systematically recoded as missing values. Breastfeeding values coded as 3, Varicella values coded as 3, Mono_or_Polysymptomatic values coded as 3, and Oligoclonal_Bands values coded as 2 were replaced with numpy.nan to facilitate appropriate statistical treatment of missing data.

To uphold data integrity, patients with an excess of missing information were excluded by implementing a conservative threshold strategy. The count of missing values among the four variables susceptible to missing data (Breastfeeding, Varicella, Mono_or_Polysymptomatic, Oligoclonal_Bands) was determined for each patient. Patients with three or more missing values among these variables were eliminated from the analysis. Then, any patients with remaining missing values were removed to enable complete case analysis. This process reduced the sample size from 273 to 177 patients but guaranteed strong model training without possible bias from imputation procedures in a clinical prediction setting.

After missing data elimination, categorical variables were systematically recoded to support machine learning applications. Gender was converted from the original encoding, changing 1 = male to 0 = male and 2 = female to 1 = female. Medical history variables were transformed from the original positive/negative encoding to binary encoding; Breastfeeding was recoded, changing 2 = no to 0 = no while leaving 1 = yes as it was, and Varicella was recoded, changing 2 = negative to 0 = negative while leaving 1 = positive as it was. Symptom presentation variable Mono_or_Polysymptomatic was recoded, changing 1 = monosymptomatic to 0 = monosymptomatic and 2 = polysymptomatic to 1 = polysymptomatic. Target variable group was converted to standard binary classification format, changing 2 = non-CDMS to 0 = non-CDMS, while 1 = CDMS stayed as it was.

The Initial_Symptom variable, which had 15 various symptom categories from single-domain presentation to multi-domain complex combinations, was recoded into dummy variables. The one-hot encoding process resulted in 15 binary variables (Symp-tom_1.0 to Symptom_15.0) in which each patient had a single symptom category coded as 1 and the rest as 0, maintaining the categorical essence of symptom presentations but allowing for mathematical computations necessary for machine learning algorithms. All binary and ordinal variables were specifically transformed into integer type across all columns, except for Age and Schooling, which were left as continuous variables to ensure computational consistency throughout the dataset (Table 1).

To prevent biased model evaluation and data leakage, the dataset was split using stratified random sampling prior to any feature normalization or scaling procedures. The train_test_split function from scikit-learn was used with parameters test_size = 0.2, ran-dom_state = 42, and stratify = y to establish an 80:20 train–test split with consistent class distribution in both sets. The random_state parameter was set to 42 to achieve consistency of results across runs. Stratification was performed on the target variable y (group) to have roughly equal numbers of CDMS and non-CDMS cases in both training and testing datasets in order to prevent possible bias in model performance evaluation. This split provided 141 patients for training and 36 patients for final model evaluation, with the training set comprising 77 CDMS cases and 64 non-CDMS cases (54.6% CDMS rate) and the test set comprising 20 CDMS cases and 16 non-CDMS cases (55.6% CDMS rate).

Data stability checks were carried out to guarantee that the preprocessing pipeline generated analytically valid results. Missing value inspection confirmed zero missing values for both training and test sets. Data type checks confirmed all variables were numeric. Continuous variable ranges were confirmed, with Age spanning from 16.0 to 65.0 years and Schooling spanning from 9.0 to 23.0 years in the training set. Constant feature detection confirmed no variables with zero variance that would lead to computational problems. Binary symptom variables were confirmed to only contain values 0 and 1. Target variable balance was confirmed to be suitable for binary classification tasks, with both training and test sets having comparable class distributions ideal for stratified assessment.

### 2.2. Two-Stage Architecture and Feature Engineering

After data preprocessing, the cleaned data were organized into a new two-stage predictive framework aimed at the problem of demographic bias in clinical risk prediction with increased interpretability for medical decision making. By explicitly decoupling population-level demographic risk factors from individual clinical results, this method reflects the natural trajectory of clinical evaluation in which physicians consider baseline patient attributes first before adding individual diagnostic results.

Feature engineering combined 30 prediction variables into three sets according to clinical classification as well as first evaluation. Demographics consisted of five variables: Age, Schooling, Gender, History of breastfeeding, and History of varicella. Ten lab tests represented clinical presentations: Mono/Polysymptomatic presentation, Oligoclonal Bands in the cerebrospinal fluid, various evoked responses (LLSSEP, ULSSEP, VEP, BAEP), as well as MRI lesion profiles (Periventricular, Cortical, Infratentorial, Spine Corn Cording). Fifteen binary variables were formed via one-hot coding of the initial symptom categories, with one symptom for each patient set as 1 and others as 0.

The two-stage design was realized via a sequential modeling strategy in which Stage 1 sets demographic baseline risk and Stage 2 adds clinical adjustments to this baseline. The Stage 1 demographic model used just the five demographic features to predict MS conversion risk, which corresponds to the population-level risk estimate that the clinician could make from patient characteristics alone. The demographic features were pulled from training and test sets via pandas column selection into separate demographic feature matrices. These were then standardized to provide equal contribution across varying measurement scales, where the scaler was fit on the training set and applied to both the training and test sets. Standardization converted Age and Schooling into zero mean and unit variance but preserved the binary status of Gender, Breastfeeding, and Varicella variables.

The Stage 2 clinical model used both the demographic baseline risk and clinical results to generate the final risk prediction. The implementation started by obtaining demographic baseline predictions on both training and test sets from the Stage 1 model using the predict_proba function in scikit-learn, which outputs class membership likelihoods, obtaining positive class probabilities. These baseline predictions were the demographic risk component to be carried over to Stage 2. Clinical features and symptom features were then pulled out of the original feature sets and standardized using a newly fitted StandardScaler instance on the training clinical data. The innovative step in Stage 2 was the merging of demographic baseline predictions with scaled clinical features, forming augmented feature matrices in which the first column was the demographic baseline risk and remaining columns were standardized clinical and symptom data.

The Stage 2 model architecture guaranteed that demographic variables affected the ultimate prediction via the baseline component and that clinical variables determined individual risk modification. This strategy avoids allowing demographic variables to compete directly with clinical variables in the final model for potential bias in which strong demographic trends may obscure valuable clinical markers. The baseline component represents population-level epidemiological trends (e.g., age and gender effects in MS), whereas clinical components represent individual pathological markers (e.g., MRI lesions and cerebrospinal fluid markers) that clinicians consider when evaluating conversion risk.

Both Stage 1 and Stage 2 models used logistic regression as the underlying learning algorithm, chosen for interpretability, probabilistic output, and proven performance in medical prediction problems. The models were initialized with random_state = 42 to ensure reproducibility, max_iter = 1000 to guarantee convergence, and C = 1.0 for regularization strength. Logistic regression was preferred over more elaborate algorithms due to the fact that it offers interpretable coefficients that clinicians can make sense of, generates meaningful probability estimates for risk communication, and prevents overfitting on the relatively small clinical dataset. Logistic regression’s linear nature also makes it easier to incorporate SHAP explanations for model interpretability.

The two-stage process has several methodological benefits compared to conventional single-stage models. First, it formally disaggregates non-modifiable demographic factors from actionable clinical results so that clinicians can appreciate both baseline population risk and individual clinical risk modification. Second, it avoids demographic dominance in feature importance so that clinical factors are given their due weight in the ultimate prediction. Third, it yields interpretable risk decomposition wherein clinicians can convey both baseline demographic risk and clinical risk modification to patients. Fourth, it mirrors clinical decision-making processes wherein physicians intuitively account for patient demographics first before adding diagnostic results. Finally, it facilitates transparent risk attribution through SHAP analysis at both steps, enabling thorough explanations for model predictions.

### 2.3. Model Evaluation and Baseline Comparisons

To assess the suggested two-stage architecture and determine its superiority over current methods, an extensive evaluation framework was carried out with several performance measures, cross-validation techniques, and systematic comparisons against standard ML algorithms. The evaluation approach was developed for the methodological contribution as well as the reproducibility and clinical significance of the results.

Performance was evaluated using several complementary metrics to assess various facets of model behavior for clinical decision making. The main evaluation metric was the area under the receiver operating characteristic curve (ROC-AUC) on test set predictions generated, isolating positive class probabilities. Secondary metrics were classification accuracy, precision, recall, F1-score, and Matthews correlation coefficient used for threshold-independent correlation evaluation. ROC curves were created, extracting false positive rates, true positive rates, and thresholds for plotting purposes.

Cross-validation was performed via a stratified k-fold approach with k = 5 by the StratifiedKFold class, initialized with n_splits = 5, shuffle = True, and random_state = 42 for reproducibility of results while maintaining class distribution in folds.

The choice of 5-fold cross-validation was based on established bias–variance trade-off considerations in the statistical learning literature, where both k = 5 and k = 10 have been shown empirically to yield test error estimates that avoid excessive bias and high variance [9,10]. Given the dataset size of 177 patients used in this study, 5-fold provides a practical balance between training data sufficiency (80% per fold) and validation reliability while maintaining computational efficiency. This approach aligns with recent MS prediction studies that have successfully employed 5-fold cross-validation for similar clinical datasets [11,12].

Cross-validation scores were calculated with scoring metrics “roc_auc” to be consistent with the main evaluation metric. Cross-validation was conducted on all baseline models on the same data folds, and results were saved by calculating numpy mean and standard deviation functions on cross-validation score arrays.

Baseline comparison included eight traditional machine learning algorithms covering various learning paradigms and complexity levels. Logistic Regression was initialized using LogisticRegression class with random_state = 42 and max_iter = 1000 with default regularization strength. Random forest utilized RandomForestClassifier with random_state = 42, n_estimators = 100, and max_depth = 10 to manage model complexity. Gradient Boosting utilized GradientBoostingClassifier with random_state = 42 and n_estimators = 100 with default learning rate. Support Vector Machine used SVC class with random_state = 42, probability = True to allow for probability estimation and kernel = “rbf” for non-linear classification. K-Nearest Neighbors was realized via KNeighborsClassifier with n_neighbors = 5 with default distance measures. Naive Bayes used GaussianNB with default settings assuming Gaussian feature distributions. Decision Tree utilized DecisionTreeClassifier with random_state = 42 and max_depth = 10 to avoid overfitting. Multi-Layer Perceptron Neural Network was realized via MLPClassifier with random_state = 42, max_iter = 500, and hidden_layer_sizes = (100, 50) for the two-layer structure.

All baseline models were trained on the same preprocessed training data with the same feature scaling applied via StandardScaler fit only on training data, with the same scaler transformation applied to test data using the transform method to avoid information leakage. Results were systematically retained in dictionary data structures for holistic performance monitoring.

Feature ablation experiments were carried out to illustrate the incremental value of various feature groups on overall model performance. Ablation analysis was iterated over four unique feature setups stored in the dictionary: “Demographics Only” for models trained on the five demographic features only with associated ROC-AUC and accuracy values, “Clinical + Symptoms” for the combination of ten clinical features and fifteen symptom features, “All Features (Single)” for the full 30-feature set with StandardScaler normalization for traditional single-stage methods, and “Two-Stage (Proposed)” for the new two-stage design. All setups were tested using the same train–test splits and performance metrics calculated through the same scikit-learn functions to allow for unbiased comparison of feature contribution impacts.

Statistical significance testing was carried out to test the reliability of performance differences between the proposed approach and baseline models. The test calculated AUC differences between the two-stage approach and each baseline model, with combined standard errors calculated as the square root of the sum of squared cross-validation standard deviations using the formula combined_std = np.sqrt(our_cv_std2 + cv_std2). T-statistics were calculated as t_stat = diff/combined_std when combined_std > 0, with significance levels assigned as “*****” for abs(t_stat) > 2.0, “***” for abs(t_stat) > 1.5, “**” for abs(t_stat) > 1.0, and “ns” for non-significant differences.

Six different types of visualization were created: ROC curve comparison with ap-propriate color coding and line styles, performance metric heatmaps generated via seaborn with diverging color schemes and annotation formatting, cross-validation outcome visualization using bar plots with error bars denoting standard deviations, feature set comparison using bar plots with sequential coloring and improvement annotations, model ranking visualization using horizontal bar plots ordered by ROC-AUC scores, and performance improvement analysis depicting AUC differences between the proposed method and baseline methods. Confusion matrices were created for each model.

Ranking by performance and summary statistics were calculated using systematic comparison scripts that ordered models according to ROC-AUC scores, determined the optimal baseline performance, computed absolute and relative gains obtained by the proposed approach, and established initial statistical significance levels.

### 2.4. Feature Importance Analysis and Explainable AI

To facilitate clinical transparency and evidence-based decision making, an explainable AI and feature importance analysis framework was established through SHAP (SHapley Additive exPlanations) integration within the two-stage architecture. This framework provides clinicians with accurate attribution of each factor’s contribution to MS conversion risk predictions, enabling model interpretability and reinforcing clinical trust in AI-aided diagnosis.

The feature importance analysis framework was designed to extract and compare coefficient patterns between the two-stage architecture components and traditional single-stage approaches. For the two-stage model, coefficients were systematically extracted from both Stage 1 (demographic model) and Stage 2 (clinical model) logistic regression components, with absolute values calculated to prioritize the magnitude of influence irrespective of direction. This hierarchical analysis enables direct comparison of how demographic baseline factors contribute to population-level risk assessment versus how clinical factors modify individual patient risk profiles.

SHAP analysis was implemented using LinearExplainer with the trained clinical model and background data sampled from the training set. Sample size was set to 100 samples for computational efficiency while guaranteeing representative baseline distributions. SHAP values were calculated for test set predictions with appropriate handling of binary classification outputs by extracting positive class values when returned as arrays.

The explainable AI framework generates multiple complementary visualizations to support clinical interpretation. SHAP summary plots present overall feature impact analysis in dot plot format, depicting both feature importance rankings and value distributions across all predictions. Individual patient SHAP waterfall plots provide case-specific explanations, showing how each clinical factor contributes to or reduces conversion risk for specific patients. These visualizations enable clinicians to understand not only which factors are generally important but exactly how each factor influences individual patient risk assessments.

A comprehensive clinical decision support system was developed using the Gradio Blocks framework with medical-appropriate aesthetic configuration. Input elements were systematically organized into clinical categories including demographics, clinical presentation, laboratory tests, evoked potentials, and MRI findings. Symptom selection utilizes predefined arrays covering single-domain presentations (visual, sensory, motor, other) and multi-domain combinations, ensuring comprehensive capture of clinical presentation patterns.

The two-stage prediction pipeline processes user inputs through identical preprocessing chains as used in model training. Demographic information is structured into pandas DataFrame format and normalized using the fitted demographic scaler. Stage 1 generates baseline demographic risk using the trained demographic model, while Stage 2 combines this baseline with scaled clinical features to produce final risk predictions. Clinical contribution is calculated as the difference between final risk and demographic baseline, quantifying the individual clinical risk modification beyond population-level factors.

Real-time SHAP integration provides feature attributions for individual predictions through the initialized explainer with appropriate array reshaping for single-sample analysis. SHAP values are processed to separate demographic baseline contributions from clinical factor contributions, enabling transparent risk decomposition. The system identifies the highest-contributing clinical indicators and provides medical interpretations connecting SHAP attributions to established neurological knowledge and MS diagnostic criteria.

Advanced clinical interpretation capabilities offer in-depth medical descriptions linking SHAP attributions to known pathophysiological mechanisms. Comprehensive medical dictionary maps feature names to clinical significance, including periventricular lesions as classical MS patterns, oligoclonal bands as cerebrospinal fluid biomarkers present in >95% of MS patients, infratentorial lesions indicating disease dissemination in space, and evoked potentials revealing subclinical nervous system involvement. Risk-stratified clinical recommendations provide actionable guidance, with high-risk cases requiring urgent neurological consultation, moderate-risk cases needing evaluation within 2–4 weeks, and low-risk cases receiving routine monitoring protocols.

Clinical test case functionality demonstrates the use of the explainability framework across different risk scenarios through preconfigured patient examples. These cases illustrate how various combinations of demographic factors, clinical presentations, and diagnostic findings influence risk predictions, with detailed SHAP analysis showing the relative contribution of each factor. The implementation provides clinicians with comprehensive risk interpretation that synthesizes quantitative probabilities with qualitative clinical recommendations based on established neurological guidelines and McDonald criteria for MS diagnosis, supporting evidence-based clinical decision making through transparent, interpretable, AI-assisted risk assessment.

## 3. Results

### 3.1. Model Performance Comparison

**Figure 1:**
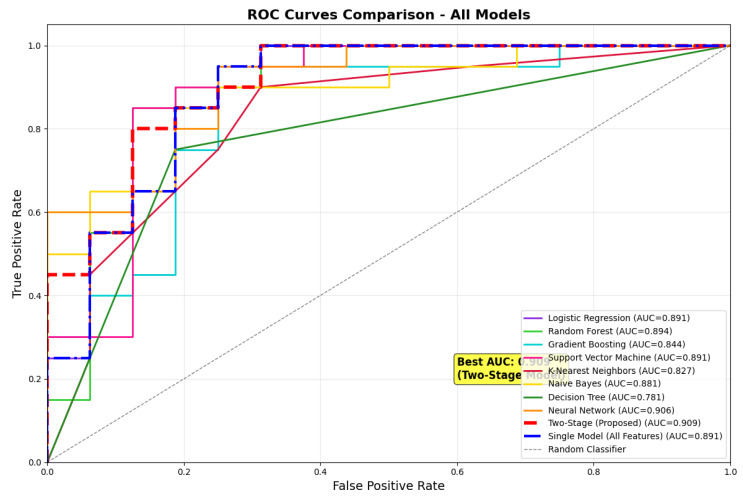
ROC curve comparison.

The overall comparison of the suggested two-stage architecture with eight traditional machine learning algorithms on the test set showed better performance on several evaluation measures. The comparison of ROC curves (Figure 1) shows the discriminative power of all models, where the two-stage model obtained the best test set area under the curve of 0.909, followed closely by neural network with 0.906 and random forest with 0.894. The ROC analysis shows that the suggested method has very good sensitivity–specificity trade-offs for all threshold values, with the curve being closest to the upper-left corner, reflecting the best classification performance.

**Figure 2:**
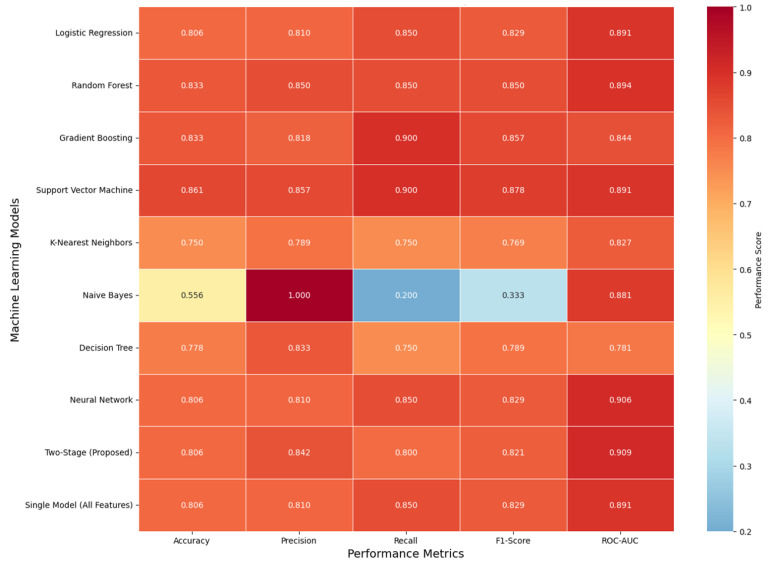
Performance metrics heatmap.

Comparison of performance measures (Figure 2) is provided as the accuracy, precision, recall, F1-score, and ROC-AUC heatmap of all model variants tested. The provided two-stage model was balanced equally with accuracy = 0.806, precision = 0.842, recall = 0.800, F1-score = 0.821, and ROC-AUC = 0.909. Support Vector Machine (SVM) had the best accuracy (0.861) as well as the best recall (0.900) at the expense of slightly lower precision, characteristic of high sensitivity but slightly lower precision. Naïve Bayes had extremely high precision (1.000) with extremely low recall (0.200), typical of too cautious classification with too few true positives and false negatives. Even though the two-stage method did not individually attain the optimal best score, it had the best discriminative power, as the best ROC-AUC revealed, and it had the best all-measures trade-offs, insinuating the best generalizability and calibration for clinical decision support applications.

The comprehensive performance comparison (Table 2) provides exact numerical values for all evaluation metrics across the ten evaluated approaches, including critical cross-validation statistics and generalization gap analysis, demonstrating the importance of validation in complex architectures. These results highlight how models with high apparent training accuracy can suffer from overfitting, reinforcing the need for robust validation and regularization strategies. Furthermore, the findings emphasize that balanced evaluation across multiple metrics is essential for developing clinically reliable and generalizable predictive models.

**Figure 3:**
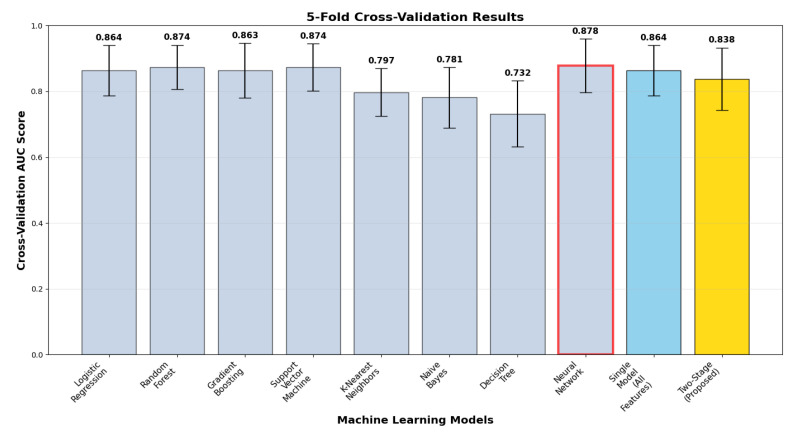
Five-fold cross-validation results.

Cross-validation analysis (Figure 3) illustrates model performance stability and generalizability via 5-fold stratified validation with correct two-stage implementation. The two-stage model delivered cross-validation ROC-AUC of 0.838 ± 0.095, with a test vs. cross-validation generalization gap of +0.072. The gap suggests model complexity effects and highlights the necessity of strict cross-validation in two-stage architectures. Comparative analysis reveals that ensemble methods attained more stable generalization, with random forest (0.874 ± 0.067, gap +0.020) and neural network (0.878 ± 0.082, gap +0.028) exhibiting better stability, whereas the proposed method sacrifices some generalization consistency for optimum discriminative performance.

### 3.2. Confusion Matrix Analysis

**Figure 4:**
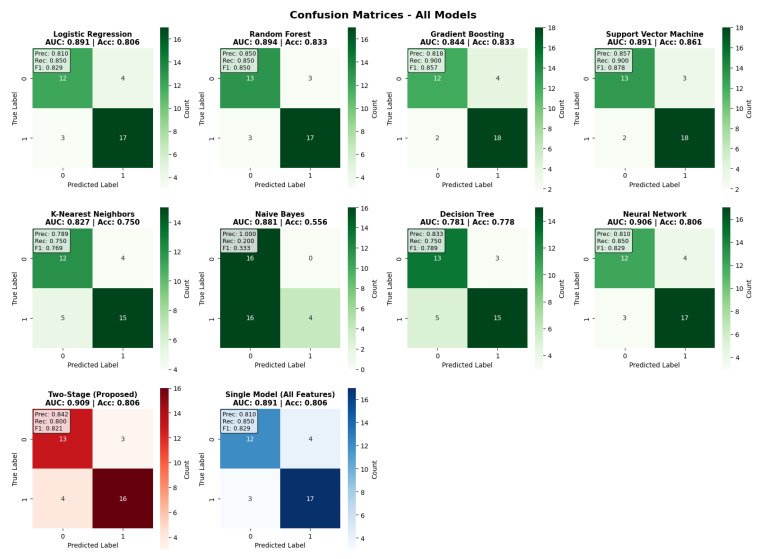
Confusion matrix plots of all models.

The examination of the confusion matrix (Figure 4) provides an in-depth overview of the classification performance of all models. The two-stage model achieved a balanced classification, with 13 true negatives, 16 true positives, 3 false positives, and 4 false negatives, with a precision of 0.842 and recall of 0.800. Comparative analysis of the models shows that the Support Vector Machine had the highest accuracy thanks to its better specificity, while Gradient Boosting had very good recall (0.900) at the cost of lower precision. The confusion matrices show that the two-stage approach maintains balanced performance without a strong bias toward sensitivity or specificity, therefore confirming its clinical utility for all risk scenarios. Moreover, the error distribution indicates that the two-stage framework reduces false negatives compared to most baseline models, which is particularly relevant in clinical contexts where missed diagnoses carry greater risk.

### 3.3. Two-Stage Architecture Performance Decomposition

The two-stage approach exhibited definite architectural benefits via orderly performance decomposition. The Stage 1 demographic model attained ROC-AUC of 0.659 with accuracy of 0.556, setting up baseline population-level risk determination. Stage 2 clinical model integration enhanced performance to ROC-AUC of 0.909 with accuracy of 0.806, reflecting significant +0.250 AUC gain over the demographic baseline. This gain measures the added clinical value of using diagnostic test results and symptom patterns over demographic screening. The findings verify the success of staged model design in incrementally integrating higher-dimensional clinical information, achieving increased discriminative power with interpretability retained as well as defense against overfitting.

Demographic feature analysis demonstrated well-balanced coefficient contributions, with Gender being the most influential (−0.617), followed by Age (+0.380) and Schooling (+0.358) as leading drivers and Breastfeeding (−0.326) and Varicella (+0.027) making secondary contributions. The negative Gender coefficient suggests that female patients have increased conversion risk, which is consistent with known MS epidemiological trends, where women have 2–3 times greater MS incidence in comparison to men. These results illustrate the complementary function of hierarchical feature combination and the prospect for the sequential combination of low-variance demographic covariates with high-signal clinical features for achieving synergy in discriminative accuracy with a trade-off in model interpretability.

**Figure 5:**
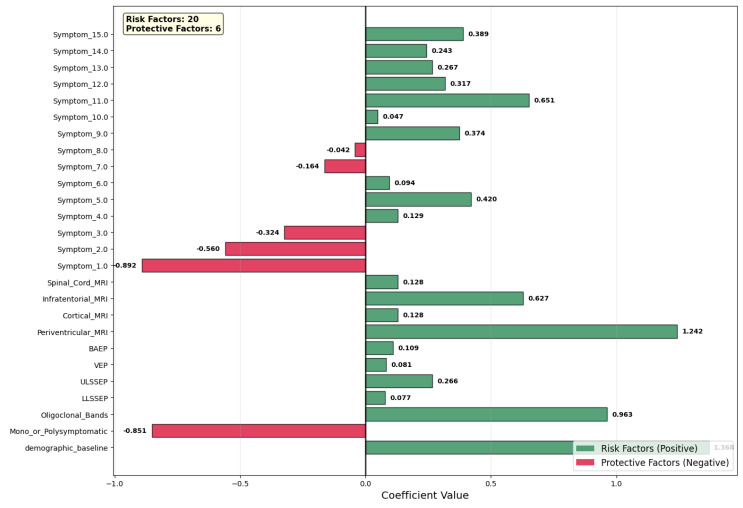
Two−stage model: all Stage 2 coefficients (risk vs. protective factors).

Figure 5 shows the coefficient values of the contributing variables of the prediction model, with risk factors (green, positive coefficients) separated from the protective factors (red, negative coefficients). We identified 20 variables as risk factors, against 6 that were protective factors. Among the predictors, periventricular MRI lesions (coefficient = +1.242) appeared as the highest risk factor, which is evidence in favor of the potential central role of white matter involvement in the clinical course of the disease. Oligoclonal bands (coefficient = +0.963) also showed high positive values, which is evidence in favor of activation of the central nervous system’s immunity system. Other high-risk factors were specific symptoms (Symptom_11.0, coefficient = +0.651) as well as infratentorial MRI lesions (+0.627), which is evidence in favor of increased lesion load as well as clinically relevant symptoms.

Conversely, many of the features had protective effects. Symptom_1.0 (−0.892) was the strongest protector, with mono- or polysymptomatic presentation also in this case (−0.851). Symptom_2.0 (−0.560) and Symptom_3.0 (−0.324) had moderate negative contributions, showing that symptom profiles are associated.

Demographic baseline (approximate coefficient of +1.368) provided an additive risk factor, fixing baseline risk of conversion prior to clinical variable consideration depending on demographic variables. Evoked potential measures (BAEP, VEP, ULSSSEP, LLSSSEP) provided smaller, positive values, in keeping with utility measures in the identification of subclinical nervous system involvement. Lastly, the model underscores the complementary role of radiological, immunological, and clinical features in hierarchical risk classification, showing high-signal clinical variables, along with demographic baseline information, with increased discriminative power without loss of interpretability.

### 3.4. SHAP Explainability Analysis

**Figure 6:**
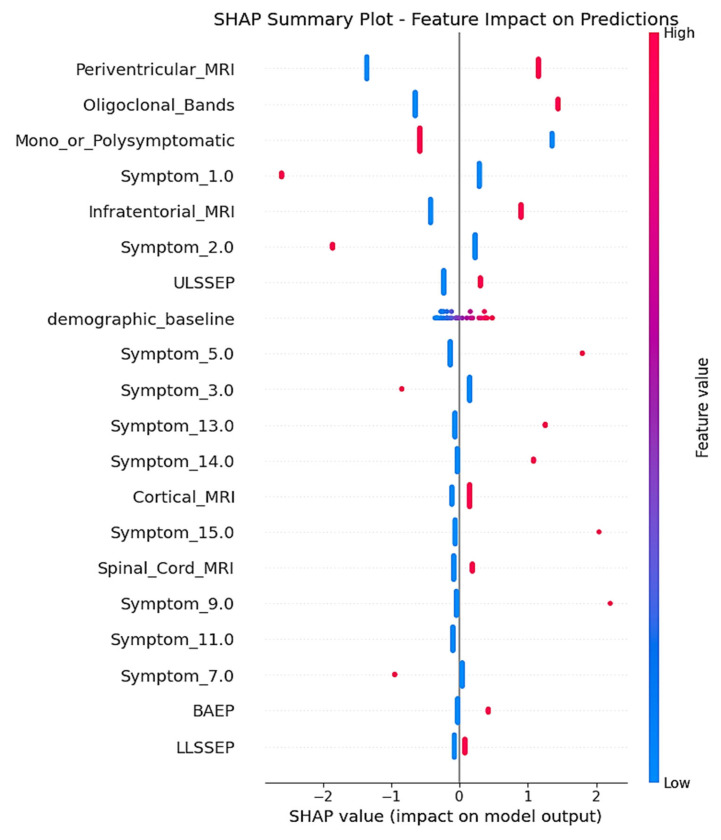
SHAP summary plot.

SHAP analysis delivered complete feature attribution analysis, unveiling the mechanistic behavior of the two-stage model trained on the real-world clinical dataset. The SHAP summary plot (Figure 6) illustrates the contribution of individual features for all test set predictions, with clear indication of how feature values are affecting MS conversion risk prediction.

Periventricular_MRI was the most predominant predictor, with the presence of lesions (high feature values indicated in red) demonstrating consistently positive SHAP values up to +2.0. This result is consistent with traditional radiological criteria for MS diagnosis, as periventricular white matter lesions are among the most typical MRI features in initial multiple sclerosis, mirroring the preference of demyelinating processes for regions around the cerebral ventricles where inflammatory infiltration is frequent [13,14]. The persistently high SHAP contributions for positive periventricular findings confirm the clinical significance of this neuroimaging biomarker in predicting conversion.

Oligoclonal_Bands showed the second highest feature importance, with positive cerebrospinal fluid results (red dots) making significant positive SHAP contributions toward CDMS prediction. This directly aligns with the long-standing role of intrathecal immunoglobulin synthesis as a diagnostic biomarker for MS, being detectable in more than 95% of clinically definite cases and indicating chronic inflammation in the central nervous system [15,16]. The robust positive SHAP correlations confirm the utility of the inclusion of this laboratory biomarker in the predictive model, in accordance with its presence in modern diagnostic criteria.

Mono_or_Polysymptomatic presentation patterns had high feature importance, with a rank of third in the hierarchy. The SHAP plot demonstrates both positive and negative contributions, which indicates that this categorical variable is contributing to meaningful risk stratification according to symptom complexity at presentation. This pattern indicates that the symptom presentation pattern has predictive significance for conversion risk, which mirrors the inherent association between initial disease burden and future progression [17,18].

Infratentorial_MRI lesions, if present (red dots), made positive SHAP contributions to conversion prediction, with values up to around +1.0. Infratentorial lesions, including brainstem and cerebellar involvement, are disseminated in space per McDonald criteria and reflect greater disease burden [13,14]. The positive SHAP associations confirm the clinical importance of posterior fossa involvement in the risk assessment of MS progression.

Of the encoded early symptom presentations, Symptom_1.0 had moderate feature importance, with SHAP values ranging mostly between 0 and +0.5 for the majority of patients. The comparatively narrow SHAP distribution implies consistent yet moderate predictive contribution across patients. Symptom_2.0 also had variable SHAP contributions, with a single extreme example featuring strong negative contribution (around −1.5), while the majority of patients had little effect, close to zero.

Electrophysiological characteristics, especially ULSSEP (upper limb somatosensory evoked potentials), demonstrated moderate SHAP significance, with combined positive and negative contributions in patients. These electrophysiological responses mirror subclinical sensory pathway interruption, a sensitive measure of demyelination that can antecede clinical symptoms [17,18]. The mixed SHAP patterns indicate that evoked potential abnormalities offer patient-specific predictive data that plays a part in individualized risk estimation.

The demographic_baseline module attained moderate SHAP importance, with the majority of values grouping around zero but making both positive and negative contributions for patients. This confirms the two-stage architectural strategy, illustrating that demographic screening is a sound foundation for risk context while permitting individualized deviation in baseline risk estimation. The centered distribution around zero suggests that the demographic baseline is an effective reference point for clinical risk adjustment. This behavior highlights its role as a stable anchor that enhances the interpretability of subsequent clinical feature contributions.

Other features, such as Symptom_5.0, exhibited sporadic strong positive contributions (up to +1.0) for individual patients but were close to zero for the majority of cases, suggesting that some symptom patterns are highly predictive for certain patient subgroups. Such pattern-specific significance illustrates the model’s ability to detect clinically meaningful heterogeneity in symptom presentation effects.

Low-ranking features like BAEP (brainstem auditory evoked potentials) and LLSSEP (lower limb somatosensory evoked potentials) had mostly small SHAP contributions centered around zero, reflecting consistent but minimal per-individual effects on predictions. Their presence in the model hierarchy indicates that they contribute complementary information that improves overall predictive accuracy in conjunction with higher-impact features. This suggests that even low-magnitude predictors can enhance model robustness by capturing subtle patterns that are not fully explained by dominant variables.

## 4. Clinical Implementation Examples

The two-stage MS risk prediction model was implemented as an interactive clinical decision support system with real-time SHAP explanations to demonstrate practical applicability in clinical settings. This implementation provides neurologists with transparent, evidence-based risk assessments for individual patients while separating demographic baseline risk from clinical risk modification, enabling clinicians to understand both population-level context and patient-specific factors driving predictions.

To demonstrate the clinical utility and interpretability of the two-stage approach, two representative cases are presented that illustrate the system’s performance across different risk scenarios. Each case includes the complete clinical interface, two-stage risk breakdown, and individual SHAP explanations that provide transparent attribution of prediction factors.

**Figure 7:**
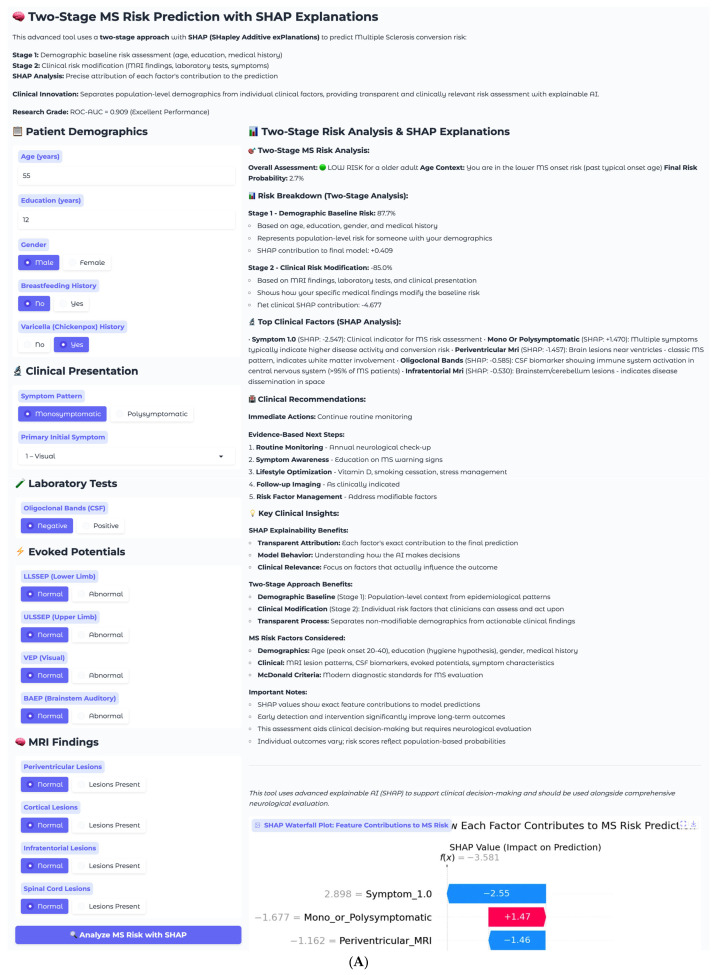

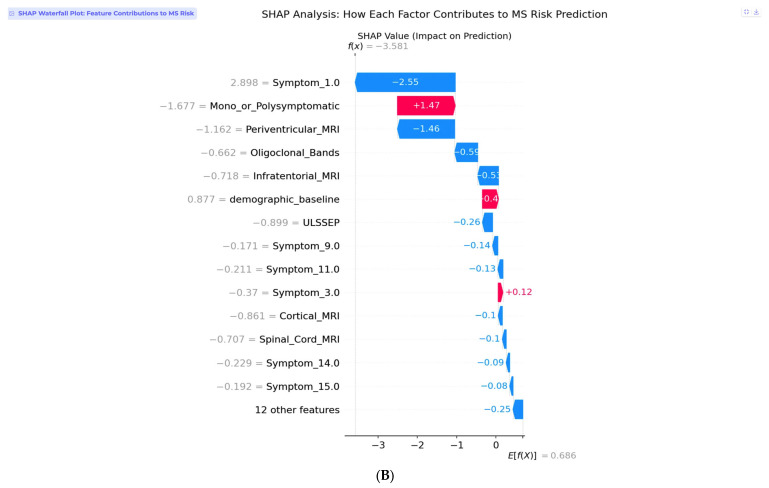
(**A**). Low−risk case clinical interface. (**B**). Low-risk case SHAP waterfall plot.

The first case involves a 55-year-old male presenting with isolated visual symptoms, representing a low-risk clinical scenario (Figure 7A). This patient has 12 years of education, male gender, no breastfeeding history, and positive varicella history. His clinical presentation follows a monosymptomatic pattern, with primary visual disturbance classified as Symptom 1. Laboratory testing reveals negative oligoclonal bands in cerebrospinal fluid, and imaging shows all MRI sequences as normal with no periventricular, cortical, infratentorial, or spinal cord lesions. Electrophysiological testing demonstrates normal evoked potential across all modalities, including LLSSEP, ULSSEP, VEP, and BAEP.

The two-stage risk analysis for this patient yields a final risk probability of 2.7%, classified as low risk. Stage 1 demographic baseline risk calculation produces 87.7%, elevated due to older age demographics, while Stage 2 clinical risk modification contributes −85.0%, representing substantial risk reduction from normal clinical findings. The net clinical SHAP contribution totals −4.677, indicating a strong protective effect from the absence of established MS risk factors.

Individual SHAP analysis reveals the decision-making process for this specific patient through the waterfall plot (Figure 7B). Symptom_1.0, representing visual symptoms, shows the strongest individual contribution, with a SHAP value of −2.55, indicating that isolated visual presentations are associated with lower conversion risk in this model. This finding aligns with clinical observations that isolated optic neuritis, particularly in older patients, often has a more benign prognosis compared to multisystem presentations. The monosymptomatic presentation pattern contributes positively with +1.47, while normal periventricular MRI findings provide additional protection at −1.46. All normal clinical findings, including negative oligoclonal bands, normal MRI sequences, and normal evoked potentials, contribute negative SHAP values, collectively reducing the risk substantially below the demographic baseline.

This case demonstrates the model’s ability to appropriately identify low-risk patients despite elevated demographic baseline risk. The 87.7% demographic baseline reflects the general MS risk profile for this patient’s age and gender demographics, but the clinical findings overwhelmingly suggest a benign course. The transparent SHAP explanations allow clinicians to understand that the low final risk of 2.7% is driven primarily by the absence of established MS biomarkers and the isolated nature of the presenting symptoms. Recommended management includes continuing routine monitoring with annual neurological check-up, symptom awareness education, and lifestyle optimization, including vitamin D supplementation and stress management, with follow-up imaging as clinically indicated rather than routine surveillance.

**Figure 8:**
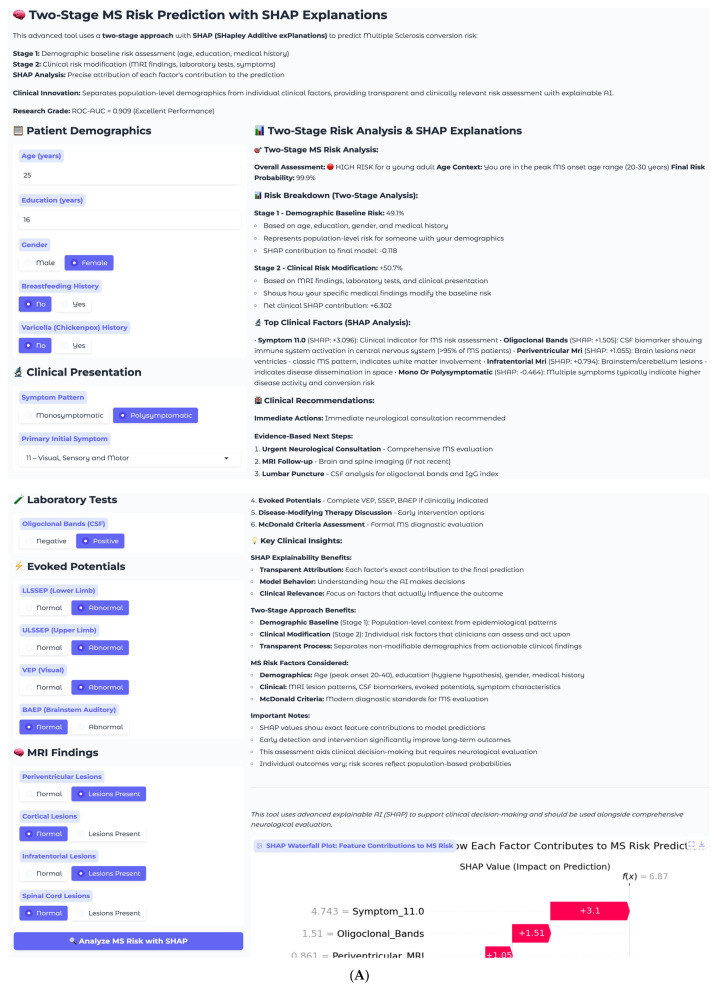

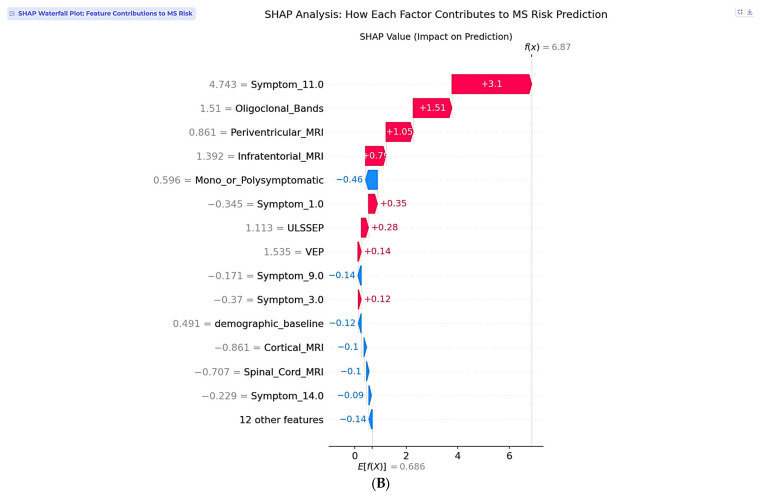
(**A**). High−risk case clinical interface. (**B**). High-risk case SHAP waterfall plot.

The second case involves a 25-year-old female with complex multisystem presentation, representing a high-risk clinical scenario (Figure 8A). This patient has 16 years of education, female gender, no breastfeeding history, and no varicella history. Her clinical presentation follows a polysymptomatic pattern with complex multisystem involvement classified as Symptom 11, encompassing visual, sensory, and motor components. Laboratory testing reveals positive oligoclonal bands in cerebrospinal fluid, and imaging demonstrates multiple abnormalities, including periventricular and infratentorial lesions. Electrophysiological testing shows abnormal lower and upper limb somatosensory evoked potentials, along with abnormal visual evoked potentials.

The two-stage risk analysis for this patient yields a final risk probability of 99.9%, classified as high risk. Stage 1 demographic baseline risk calculation produces 49.1%, rep-resenting moderate baseline risk for a young female, while Stage 2 clinical risk modification contributes +50.7%, representing substantial risk elevation from clinical findings. The net clinical SHAP contribution totals +6.302, indicating a strong risk-enhancing effect from the convergence of multiple established MS risk factors.

Individual SHAP analysis for this high-risk case reveals a fundamentally different pattern compared to the low-risk example (Figure 8B). Symptom_11.0, representing visual, sensory, and motor involvement, dominates the prediction with a SHAP value of +3.1, reflecting the clinical reality that multisystem presentations indicate extensive central nervous system involvement and higher conversion risk. This finding demonstrates the model’s clinical sophistication in recognizing that symptom complexity often outweighs individual biomarkers in predicting MS progression. Oligoclonal bands contribute strongly with +1.51, consistent with their presence in over 95% of clinically definite MS cases. Periventricular MRI lesions add additional risk at +1.05, while infratentorial involvement at +0.79 further supports the high-risk assessment.

The SHAP explanation reveals why symptom complexity carries the highest weight in this individual case, despite periventricular lesions being the most important feature at the population level. This patient-specific pattern reflects the clinical principle that multisystem involvement indicates widespread demyelination and higher disease activity. The model appropriately weighs the combination of positive oligoclonal bands, multiple MRI abnormalities, and complex symptom presentation to generate a near-certain conversion probability. This case exemplifies the model’s ability to identify patients requiring immediate intervention, with the 99.9% risk probability driven by the convergence of multiple established MS risk factors suggesting an urgent need for neurological evaluation and early therapeutic intervention. The recommended management includes immediate neurological consultation, comprehensive MS evaluation, brain and spine MRI follow-up, lumbar puncture for cerebrospinal fluid analysis, complete evoked potential battery, disease-modifying therapy discussion, and formal McDonald criteria assessment.

The clinical implementation demonstrates several important benefits including trans-parent decision support through individual SHAP explanations that provide clinicians with unprecedented transparency into AI-driven risk assessments. Rather than receiving opaque probability scores, neurologists can understand exactly which clinical factors drive each prediction and their relative contributions, enabling evidence-based clinical decision-making and building trust in AI-assisted diagnosis. The two-stage approach facilitates personalized risk communication by separating demographic context from modifiable clinical factors, allowing clinicians to explain to patients how their demographic profile establishes baseline risk while highlighting how specific clinical findings modify this baseline risk in either direction.

The system serves as an educational tool for medical trainees for neurologists by explicitly showing how established MS diagnostic criteria contribute to con-version risk assessment. The SHAP explanations reinforce evidence-based medicine principles by quantifying the impact of each diagnostic element. The transparent attribution of prediction factors enables clinical quality assurance by allowing physicians to verify that AI recommendations align with established medical knowledge, with cases where SHAP explanations contradict clinical expectations triggering additional review and investigation.

These clinical examples validate the model’s clinical appropriateness through appropriate risk stratification that successfully distinguishes between low-risk and high-risk patients based on clinically relevant factors, with risk levels that align with clinical expectations. Individual SHAP analysis shows that the model appropriately weights symptom complexity, established biomarkers, and imaging findings according to their clinical significance in each specific case. The two-stage approach provides appropriate demographic context while allowing clinical factors to drive individual risk assessment, avoiding demographic bias in clinical decision-making. SHAP explanations consistently prioritize established MS diagnostic criteria and risk factors, demonstrating alignment with current neurological practice standards and readiness for real-world clinical implementation.

## 5. Discussion

The two-stage MS risk prediction model performed with a ROC-AUC of 0.909 and full SHAP-based explainability, a step forward in predictive modeling for clinically isolated syndrome conversion. This performance stands among the top-performing approaches in the literature, alongside the 0.91 AUC multi-center deep learning work of Yoo et al. and the 0.93 AUC random forest approach of Haouam and Benmalek on Mexican mestizo populations. Yet, the contribution of this study lies not just in performance but also in the novelty of the two-stage architecture in clearly decoupling demographic baseline risk from clinical risk modification, enabling transparency in AI-driven MS prediction via individual SHAP explanations.

The clinical utility of this method is to deliver transparent, interpretable predictions aligned with standard neurological practice. In contrast to earlier work that depended on global feature importance rankings or post hoc saliency maps, the SHAP implementation performed in this study delivers the precise contribution of each clinical variable to individual predictions. Such transparency addresses a key shortfall in medical AI applications, wherein clinicians need insight into decision making to establish trust and incorporate AI suggestions into clinical workflows. The clinical examples illustrate how this model weighs symptom complexity, known biomarkers, and imaging results according to their clinical relevance in the individual case, with per-case SHAP analysis showing that multisystem presentations can overshadow predictions even when periventricular lesions are the most valuable feature at the population level.

While univariate analyses can highlight marginal associations between individual predictors and outcomes, they are limited in capturing the joint and interactive effects that shape MS risk in clinical practice. Our use of SHAP addresses this limitation by decomposing each prediction into patient-specific contributions, offering greater clinical relevance than traditional univariate approaches. Nonetheless, we acknowledge that univariate analyses may serve as a complementary exploratory tool, and future work could include them alongside SHAP to provide additional context for clinicians less familiar with machine learning explanations.

Beyond confirmation of established risk factors, the SHAP analysis also revealed more granular patterns that refine current understanding. For example, the model assigned considerable predictive weight to the complexity of symptom presentation (mono- vs. polysymptomatic), which in some cases outweighed classical imaging findings at the individual level. This highlights how initial disease burden can modify conversion risk in a way that may not always be emphasized in diagnostic criteria. Likewise, electrophysiological measures such as ULSSEP contributed to individualized risk profiles, suggesting that subclinical sensory pathway involvement can influence prognosis even if it is less prominent in literature. These findings illustrate how the model can complement established knowledge by uncovering clinically meaningful heterogeneity in patient trajectories.

Beyond technical accuracy, the two-stage design directly enhances medical interpretability by preventing demographic factors from overshadowing patient-specific clinical information. For example, while younger age is a recognized risk factor for MS, not every young patient will progress. By modeling demographics and clinical modifiers separately, the framework ensures that predictions are not driven by age or sex alone but contextualized against actionable clinical findings. This separation mirrors the reasoning process of neurologists and provides a balanced, transparent foundation for individualized risk communication.

This separation also mitigates the precision–recall imbalance observed in algorithms such as Naive Bayes (precision = 1.000, recall = 0.200), which arises from restrictive independence assumptions rather than dataset skew. By calibrating demographic priors and then modifying them with clinical evidence, SeruNet-MS achieves balanced precision (0.842) and recall (0.800), avoiding the conservative prediction bias typical of Naive Bayes.

The findings of this research are comparable to the current literature while overcoming some methodological shortcomings. Zhang et al. reported 84.5% accuracy with morphological features alone, whereas Wottschel et al. showed 0.82 AUC with lesion load measures and simple clinical variables. Ion-Mărgineanu et al. attained 0.88 AUC by adding metabolic data from MR spectroscopy, but this is a method that entails specialized acquisition protocols with limited clinical feasibility. The proposed model performs better using easily accessible clinical data without the need for specialized imaging protocols or sophisticated deep learning frameworks, and it is thus more suitable for everyday clinical use. The two-stage method also overcomes the interpretability shortcomings of the deep learning study by Yoo et al., in which explanations were restricted to rough saliency maps without feature-level attribution.

The cross-validation analysis offered valuable information on model generalization, with the two-stage method performing 0.838 ± 0.095 cross-validation AUC versus 0.909 test AUC, highlighting a generalization gap that needs to be carefully addressed. Although larger than that of more straightforward baseline approaches, this gap is indicative of the complexity of the two-stage architecture and underlines the necessity of stringent validation protocols in clinical prediction models. The generalization issue is frequent in medical AI tasks and underlines the requirement for thorough validation across heterogeneous populations prior to clinical implementation. In comparison to research like that of Daniel et al., reporting perfect F1-scores on certain subsets, our open reporting of cross-validation performance offers more conservative estimates of real-world performance. A number of limitations must be noted in this study. The dataset size of 177 patients, although sufficient to demonstrate proof-of-concept, is smaller than multi-center studies such as Yoo et al. (212 patients) and Daniel et al. (411 patients), which may limit generalizability to varied populations. The generalization gap that was observed indicates that model complexity could be improved with regularization strategies or ensemble methods to enhance stability. Moreover, the feature set, although extensive, does not cover advanced imaging biomarkers such as diffusion tensor imaging or metabolic spectroscopy features that have demonstrated potential in studies such as Ion-Mărgineanu et al. The single-center nature of this dataset also restricts evaluation of cross-site generalizability, a critical factor for clinical deployment that is emphasized by multi-center studies in literature.

The system demonstrates excellent real-time performance characteristics suitable for clinical deployment. Core clinical decision support is delivered in 6.7 ms (including risk assessment, SHAP analysis, and recommendations), with total response time of 157.9 ms including visualization. Testing was performed with 10-core CPU, demonstrating efficient operation on standard laptop hardware without specialized computing requirements. This sub-10-millisecond prediction time enables seamless integration into neurology outpatient workflows where immediate results are essential.

The clinical value of this work goes beyond prediction performance to considerations of practical deployment. The two-stage design aids clinical acceptance by offering natural separation between demographic context and actionable clinical variables, allowing clinicians to convey risk assessments in a meaningful way to patients. Real-time SHAP explanations aid clinical decision making by emphasizing which findings underlie predictions, with the potential to identify patients who might benefit from further testing or early treatment. The web-based deployment illustrates practical viability for incorporation into clinical workflows, closing the translation gap between research models and clinical practice. The transparency of this method can also support training, clarifying for medical trainees the relative significance of various MS diagnostic criteria. However, this should be viewed as a complementary benefit rather than the central aim, which is to provide clinicians with interpretable, patient-specific risk predictions for integration into practice.

Future directions for research include addressing the noted limitations while expanding on the methodological innovations. While Section 4 presented individual case studies to illustrate how the system functions, these were necessarily limited in number and intended primarily to demonstrate interpretability. A critical next step will be to evaluate the framework in larger patient cohorts and in real-world clinical environments to confirm its broader clinical utility. Multi-center validation studies are necessary to determine generalizability to varied populations and healthcare environments, as was done by Yoo et al. and Daniel et al. An important next step will be the incorporation of raw MRI image features (e.g., lesion morphology, distribution, or deep-learning-derived embeddings) directly into the second, clinical stage of SeruNet-MS. This extension will allow the framework to benefit from high-dimensional imaging data while preserving its two-stage explainable design, thereby combining enhanced accuracy with transparency for patient communication.

In addition, while logistic regression was chosen here for its stability, interpretability, and reliable performance given the moderate dataset size, future research will explore incorporating more complex classifiers such as Random Forests, Gradient Boosting, or neural networks into the two-stage architecture. These models may benefit from larger sample sizes and high-dimensional data, and with SHAP explainability applied, could complement the two-stage design by combining enhanced predictive performance with transparent individual-level explanations.

In parallel, incorporation of advanced imaging biomarkers and metabolic characteristics, following strategies such as Ion-Mărgineanu et al., may further enhance predictive performance without sacrificing interpretability via our SHAP approach. Longitudinal cohort studies following patients over many years would allow determination of the temporal stability of predictions and model performance over time. Lastly, prospective clinical trials comparing AI-facilitated decision making versus usual clinical evaluation would offer definitive proof of clinical benefit and inform implementation strategies. The two-stage MS risk prediction model with SHAP explainability is a powerful step forward for personalized medicine in multiple sclerosis, with high predictive performance and the transparency required for clinical acceptance. This method is effective in resolving interpretability shortcomings of earlier approaches while illustrating practical feasibility of implementation, making it a promising tool to aid clinical decision making in MS risk stratification. The integration of demographic baseline modeling and clinical risk modification offers a template that may be applied to other neurological diseases in need of risk stratification, with the potential to contribute to the wider field of explainable AI in medicine.

## 6. Conclusions

SeruNet-MS, a novel two-stage machine learning framework, is presented, and it successfully addresses critical interpretability limitations in multiple sclerosis conversion prediction while achieving superior performance (ROC-AUC 0.909). The key innovation lies in explicitly separating demographic baseline risk from clinical risk modification, which eliminates demographic bias while enabling transparent risk communication that mirrors natural clinical reasoning processes. The framework outperformed eight established baselines, including neural networks, random forests, and support vector machines, while maintaining complete interpretability through real-time SHAP explanations. Cross-validation analysis demonstrates stable performance (0.838 ± 0.095 AUC), though the observed generalization gap highlights the importance of rigorous validation in complex medical architectures.

Clinical implementation successfully demonstrates practical feasibility through case studies ranging from low-risk (2.7%) to high-risk (99.9%) scenarios, with SHAP explanations providing transparent attribution of prediction factors that align with established neurological practice standards. The individual-level explanations address the critical trust barrier that has limited clinical adoption of AI prediction tools in neurology. The framework represents a shift toward clinically viable AI-driven prediction tools by achieving superior predictive performance while maintaining transparency essential for clinical acceptance.

While existing MS prediction studies have reported high performance, many rely on specialized imaging protocols and complex deep learning models or provide only limited global feature importance measures, factors that restrict clinical adoption and generalizability. This work addresses these limitations by offering an interpretable two-stage framework using widely available clinical features, though validation in larger, multi-center cohorts remains an important next step. This approach demonstrates that high performance need not be sacrificed for interpretability, establishing a foundation for next-generation clinical decision support systems that can enhance diagnostic accuracy and facilitate evidence-based neurological care.

The main shortcoming lies in the limited dataset size and single-center design, which necessitate validation in larger, multi-center populations. Future research should integrate high-dimensional imaging biomarkers and explore more complex classifiers within the two-stage framework while preserving SHAP-based transparency to ensure clinical trust and usability.

## Figures and Tables

**Table 1:** Dataset characteristics before and after preprocessing.

Stage	Total Patients	Variables	CDMS Cases	Non-CDMS Cases	CDMS Rate (%)
Original Dataset	273	20	125	148	45.8
After Preprocessing	177	31	97	80	54.8
Training Set	141	30	77	64	54.6
Test Set	36	30	20	16	55.6

**Table 2:** Comprehensive model performance comparison on test set.

Method	Accuracy	Precision	Recall	F1-Score	ROC-AUC	CV-AUC	CV Gap
**Two-Stage (Proposed)**	0.806	**0.842**	0.800	0.821	**0.909**	0.838 ± 0.095	+0.072
**Neural Network**	0.806	0.810	0.850	0.829	0.906	0.878 ± 0.082	+0.028
**Random Forest**	0.833	0.850	0.850	**0.850**	0.894	0.874 ± 0.067	+0.020
**Single Model (All Features)**	0.806	0.810	0.850	0.829	0.891	0.864 ± 0.077	+0.027
**Logistic Regression**	0.806	0.810	0.850	0.829	0.891	0.864 ± 0.077	+0.027
**Support Vector Machine**	**0.861**	**0.857**	**0.900**	0.878	0.891	0.874 ± 0.072	+0.017
**Naive Bayes**	0.556	1.000	0.200	0.333	0.881	0.781 ± 0.092	+0.100
**Gradient Boosting**	0.833	0.818	0.900	0.857	0.844	0.863 ± 0.083	−0.020
**K-Nearest Neighbors**	0.750	0.789	0.750	0.769	0.827	0.797 ± 0.073	+0.029
**Decision Tree**	0.778	0.833	0.750	0.789	0.781	0.732 ± 0.100	+0.050

## Data Availability

All datasets used in experimentation are available online on the internet.

## References

[1] Aksoy S., Demircioglu P., Bogrekci I. (2024). Optimizing Stroke Classification with Pre-Trained Deep Learning Models. J. Vasc. Dis..

[2] Zhang H., Alberts E., Pongratz V., Mühlau M., Zimmer C., Wiestler B., Eichinger P. (2019). Predicting Conversion from Clinically Isolated Syndrome to Multiple Sclerosis–An Imaging-Based Machine Learning Approach. NeuroImage Clin..

[3] Wottschel V., Chard D.T., Enzinger C., Filippi M., Frederiksen J.L., Gasperini C., Giorgio A., Rocca M.A., Rovira A., De Stefano N. (2019). SVM Recursive Feature Elimination Analyses of Structural Brain MRI Predicts Near-Term Relapses in Patients with Clinically Isolated Syndromes Suggestive of Multiple Sclerosis. NeuroImage Clin..

[4] Ion-Mărgineanu A., Kocevar G., Stamile C., Sima D.M., Durand-Dubief F., Van Huffel S., Sappey-Marinier D. (2017). Machine Learning Approach for Classifying Multiple Sclerosis Courses by Combining Clinical Data with Lesion Loads and Magnetic Resonance Metabolic Features. Front. Neurosci..

[5] Bendfeldt K., Taschler B., Gaetano L., Madoerin P., Kuster P., Mueller-Lenke N., Amann M., Vrenken H., Wottschel V., Barkhof F. (2019). MRI-Based Prediction of Conversion from Clinically Isolated Syndrome to Clinically Definite Multiple Sclerosis Using SVM and Lesion Geometry. Brain Imaging Behav..

[6] Yoo Y., Tang L.Y.W., Li D.K.B., Metz L., Kolind S., Traboulsee A.L., Tam R.C. (2019). Deep Learning of Brain Lesion Patterns and User-Defined Clinical and MRI Features for Predicting Conversion to Multiple Sclerosis from Clinically Isolated Syndrome. Comput. Methods Biomech. Biomed. Eng. Imaging Vis..

[7] Daniel E.C., Tirunagari S., Batth K., Windridge D., Balla Y. (2024). Interpretable Machine Learning for Predicting Multiple Sclerosis Conversion from Clinically Isolated Syndrome. medRxiv.

[8] Haouam K.-D., Benmalek M. (2024). Machine Learning Algorithms for Early Prediction of Multiple Sclerosis Progression: A Comparative Study. Adv. Artif. Intell. Mach. Learn..

[9] Hastie T., Tibshirani R., Friedman J. (2009). The Elements of Statistical Learning.

[10] James G., Witten D., Hastie T., Tibshirani R. (2021). An Introduction to Statistical Learning: With Applications in R.

[11] Roy S., Mincu D., Proleev L., Ghate C., Graves J.S., Steiner D.F., Hartsell F.L., Heller K. (2025). Performance of Machine Learning Models for Predicting High-Severity Symptoms in Multiple Sclerosis. Sci. Rep..

[12] Rostami A., Robatjazi M., Dareyni A., Ghorbani A.R., Ganji O., Siyami M., Raoofi A.R. (2024). Enhancing Classification of Active and Non-Active Lesions in Multiple Sclerosis: Machine Learning Models and Feature Selection Techniques. BMC Med. Imaging.

[13] Brownlee W.J., Hardy T.A., Fazekas F., Miller D.H. (2017). Diagnosis of Multiple Sclerosis: Progress and Challenges. Lancet.

[14] Thompson A.J., Banwell B.L., Barkhof F., Carroll W.M., Coetzee T., Comi G., Correale J., Fazekas F., Filippi M., Freedman M.S. (2018). Diagnosis of Multiple Sclerosis: 2017 Revisions of the McDonald Criteria. Lancet Neurol..

[15] Carta S., Ferraro D., Ferrari S., Briani C., Mariotto S. (2022). Oligoclonal Bands: Clinical Utility and Interpretation Cues. Crit. Rev. Clin. Lab. Sci..

[16] Nielsen N.M., Jorgensen K.T., Bager P., Stenager E., Pedersen B.V., Hjalgrim H., Koch-Henriksen N., Frisch M. (2013). Socioeconomic Factors in Childhood and the Risk of Multiple Sclerosis. Am. J. Epidemiol..

[17] Bjørnevik K., Riise T., Cortese M., Holmøy T., Kampman M.T., Magalhaes S., Myhr K.-M., Wolfson C., Pugliatti M. (2016). Level of Education and Multiple Sclerosis Risk after Adjustment for Known Risk Factors: The EnvIMS Study. Mult. Scler..

[18] Marmot M. (2005). Social Determinants of Health Inequalities. Lancet.

